# Diastolic Dysfunction and Renal Disease: Analysis, Mechanisms, and Different Perspectives

**DOI:** 10.7759/cureus.76959

**Published:** 2025-01-05

**Authors:** Joana Araújo

**Affiliations:** 1 Family Medicine, Unidade Local de Saúde do Alto Minho, Viana do Castelo, PRT

**Keywords:** chronic kidney disease, diastolic dysfunction, heart failure, phosphocalcium metabolism, vitamin d

## Abstract

Over the past few decades, heart failure with preserved ejection fraction has established itself as an individual clinical entity. Although it is associated with a better prognosis, it offers high resistance to classic treatment techniques, and the frequency of hospitalizations and mortality rates are comparable to cases of heart failure with reduced ejection fraction. Heart failure often leads to death and morbidity, and there has recently been a growing interest in studying the relationship between cardiac and renal function due to epidemiological evidence indicating that even a modest deterioration in renal function is a considerable risk factor in patients with heart failure, myocardial infarction or in the context of cardiovascular surgery. In fact, studies have proven that patients with chronic kidney disease have a cardiovascular risk about 10 times higher than a population of the same age, sex, and race without it.

Before writing this review, research literature on heart failure with preserved ejection fraction and chronic kidney disease was reviewed.

Studies have shown that in patients with chronic kidney disease, heart failure is mostly caused by the presence of left ventricular diastolic dysfunction, with aggravating comorbidities such as high blood pressure and coronary heart disease. A possible underlying mechanism may be the excessive activation of the renin-angiotensin-aldosterone system, which is known to be a determinant in the onset of profibrotic factors. In fact, it is known that, in patients with chronic heart failure, the renin-angiotensin-aldosterone system is activated, and it has even been shown that the activity of increased plasma renin levels directly contributes to mortality. Angiotensin II promotes cardiac remodeling, and aldosterone may increase myocardial fibrosis, which is a marker of diastolic dysfunction and cardiac necrosis, acting as an endogenous bioactive factor involved in the process of vascular calcification. On the other hand, the development of diastolic dysfunction in patients with chronic kidney disease may result from disorders of metabolism. Besides, evidence indicates that individuals with 25-hydroxyvitamin D deficiency have an increased risk of developing various cardiovascular conditions, such as hypertension, peripheral vascular disease, myocardial infarction, diabetes mellitus, heart failure, and even death. In recent studies, it has been described that the direct effect of vitamin D on cardiomyocytes consists essentially in the acceleration of myocardial relaxation, leading to the hypothesis that it causes a determining effect on diastolic function.

Currently, both heart failure with preserved ejection fraction and chronic kidney disease are very prevalent and are closely linked to several other factors, including disturbances in phospho-calcium metabolism and variations in serum vitamin D levels. Although the concept of heart failure began to be explored a few decades ago, further studies are required in order to explain the factors that created the controversy behind the concept of diastolic dysfunction. This review aims precisely to identify the areas that lack further investigation, which can be essential to the development of more effective treatments and subsequently obtain better outcomes.

## Introduction and background

Over the last few decades, heart failure with preserved ejection fraction has claimed itself as an individual clinical entity [1], and these cases of heart failure have a more marked persistence in time following ischemic episodes triggered by stress (induced by physical exercise) [2]. Although it is associated with a better prognosis, the frequency of hospitalizations and mortality rates in these cases are comparable to cases of heart failure with reduced ejection fraction [3]. In fact, although the prognosis in cases of indiscriminate heart failure has been improving in the aforementioned period of time, the same is not true for the specific case of diastolic dysfunction, which offers high resistance to classical treatment techniques [4]. Heart failure often leads to death and morbidity. Recently, there has been a growing interest in studying the relationship between cardiac and renal function due to epidemiological evidence showing that even a modest deterioration in renal function works as an important risk factor for patients with heart failure, myocardial infarction, or in the context of cardiovascular surgery. Moreover, the level of renal function is an independent cardiovascular risk factor. Studies have proven that patients with chronic kidney disease (CKD) have a cardiovascular risk about 10 times higher than a population of the same age, sex, and race without the disease [5].

## Review

Diastolic function

Each cardiac cycle includes a phase of contraction and ejection (systole) and relaxation with a filling of the heart cavities (diastole). Regarding the parameters that determine diastolic function, it is worth highlighting myocardial relaxation - conditioned by load, cardiomyocyte inactivation associated with calcium homeostasis and regulators of the myofilament cross-bridge cycle; the passive properties of the ventricular walls - conditioned by the rigidity of the myocardium, the thickness of the walls and the geometry of the ventricular chambers themselves; and extrinsic factors to the heart itself, namely structures surrounding the ventricles (pericardium, lungs, left atrium, pulmonary veins and mitral valve) and heart rate [6]. Relaxation is the process that aims to return the myocardium to a state of strength and length, not under stress, encompassing the majority of the ventricular ejection period and the initial part of the rapid filling phase. The drop in pressure at the left ventricle is the hemodynamic manifestation of ventricular relaxation [7]. If ventricular relaxation is faster, lower ventricular pressures will be reached, which will facilitate the passage of blood through the atria. Taking this premise into account, diastolic function can be evaluated by monitoring the pressure drop in the left ventricle, namely by determining the time it takes for the ventricle to relax isovolumetrically (isovolumetric relaxation, time), the maximum pressure drop velocity (dP/dtminimum) and the Tau time constant (obtained by the slope of the pressure curve). 

Impaired diastolic function is accompanied by increased filling pressures and heart failure with preserved ejection fraction (HFpEF) [8]; that is, it corresponds to a disturbance in ventricular relaxation, distensibility, or filling with normal or altered ejection fraction in the presence or absence of symptoms. Characteristically, in this disorder, there is a preserved telesystolic pressure-to-volume relationship and ejection volume and an increase in filling pressures. It is common for these patients to manifest pulmonary edema (due to the direct influence of increased passive rigidity), dyspnoea (subsequent to the increased breathing effort, as a compensating mechanism, due to the reduced lung compliance caused by high filling pressures), and chronic intolerance to physical exercise, with episodes of dyspnoea and fatigue associated with the effort underlying both increased left ventricular filling pressures and progressive increase in diastolic dysfunction [9]. Impaired cardiac relaxation is a determining factor in the onset of symptoms in patients with underlying chronic heart failure, and simultaneously, factors such as age, diabetes mellitus, aging, and ischemia correlate positively with the prevalence of diastolic dysfunction [10]. Interestingly, diastolic cardiac dysfunction is associated with migraines, and, in fact, a long history of migraine attacks is an independent factor in predicting the development of diastolic dysfunction [11]. 

The definition of HFpEF has been evolving over time. HFpEF is now defined as heart failure (HF) with left ventricular ejection fraction (LVEF) >50% in the absence of prior reduced LVEF [12]. Therefore, most guidelines define HFpEF clinically as (1) the presence of symptoms and signs of HF; (2) an LVEF ≥50%; (3) careful exclusion of HFpEF mimickers; and (4) evidence of elevated LV filling pressure or non-invasive correlates (elevated E: e' ratio, increased left atrial volume, elevated natriuretic peptides (NP)) [12,13]. It is estimated that 50% of all patients with heart failure have HFpEF [14]. Treatment of HFpEF includes both lifestyle-based therapy and medical therapy. A combination of a balanced, healthy diet and regular exercise has an additive effect [15]. Regarding medical treatment, management of risk factors and comorbidities should first be performed [12]. Diuretics play an important role in the management of HFpEF as they relieve symptoms caused by volume overload [12]. If comorbidities such as coronary artery disease or atrial fibrillation are present, Beta-blockers are often used [12]. Renin-angiotensin-aldosterone system (RAAS) inhibitors and mineralocorticoid receptor antagonists (MRAs) have a less effective effect in treating patients with HFpEF because as LVEF increases, it seems the RAAS has less impact in the pathophysiology of HF [16]. Another pharmacological option is angiotensin receptor neprilysin inhibitor (ARNI) sacubitril-valsartan. Despite apparently less effective, response to ARNI treatment may vary according to HFpEF phenotypes [17]. At last, sodium-glucose cotransporter 2 inhibitor (SGLT2i) seem to play a valuable role in the achievement of better outcomes in HFpEF [18]. The "EMPEROR-Preserved" study was performed to evaluate the effects of SGLT2 inhibition with empagliflozin on major HF outcomes in patients with HFpEF [18]. Differences between the placebo and empagliflozin groups for the primary outcome were assessed for statistical significance at an alpha level of 0.0497, with adjustment for age, sex, geographic region, diabetes status, left ventricular ejection fraction, and eGFR [18]. Treatment effects were expressed as hazard ratios with 95% confidence intervals [18].

Cardio-renal syndrome

The kidney plays a critical role in the maintenance of the body's homeostasis, one of its functions being the regulation of extracellular fluid volume. Actually, even small changes in renal function can disturb that balance. It is known that, in patients with chronic heart failure, even a moderate reduction in glomerular filtration rate is associated with a poor prognosis [19], something already measured by clinical observations suggesting that the rate of sudden death in patients with combined cardio-renal dysfunction is increased [20]. Several hypotheses have been postulated that may corroborate this cardio-renal interaction, namely endothelial dysfunction, inflammation, imbalance between nitric oxide and reactive oxygen species, or activation of the sympathetic nervous and renin-angiotensin-aldosterone systems [21]. Renal retention of water and sodium is a triggering factor for heart failure associated with hypervolemia, and the use of diuretics is common in the treatment of patients with dyspnea and tachycardia [22]. In cases of persistent overload of extracellular fluid volume, there is usually a progression of heart failure explained by several mechanisms triggered by this excessive volume (dilation of the heart cavities, increase in left ventricular mass, weakening of the atriculorenal reflexes). 

Chronic renal failure is defined as the presence of kidney damage or an estimated glomerular filtration rate (eGFR) less than 60ml/min/1.73 m^2^ for at least three months, and it correlates with a number of interconnected factors (hypertension, chronic volume overload, anemia and metabolic factors such as acidosis, hypoxia, hypocalcemia and high levels of parathyroid hormone [23]. There are many other risk factors such as increased oxidative stress, inflammation associated with increased levels of C-reactive protein (CRP), phosphate retention, and changes in cardiac morphology, namely left ventricular hypertrophy, advanced coronary atherosclerosis, microvascular disease and diffuse interstitial fibrosis of the myocardium. According to international registries, the leading cause of death in patients with advanced chronic kidney disease (stages 4-5, eGFR < 30 mL/min/1.73 m^2^) is cardiovascular disease [24], to which the aspects listed above contribute. It has been shown in studies that, in patients with chronic kidney disease, heart failure is mostly caused by the presence of left ventricular diastolic dysfunction with aggravating comorbidities such as high blood pressure and coronary heart disease [24,25]. Echocardiographic studies have revealed that left ventricular diastolic dysfunction in patients with CKD is relatively common [26]. In the CRIC study (CKD stage 2-4), diastolic function was impaired in 71% of patients [27], while another study found that patients with CKD have a high incidence of structural cardiac abnormalities and diastolic dysfunction [28]. A possible underlying mechanism may be the excessive activation of the renin-angiotensin-aldosterone system, which is known to be a determinant of the onset of profibrotic factors. In fact, it is known that, in patients with chronic heart failure, the renin-angiotensin-aldosterone system is activated, and it has even been shown that the activity of increased plasma renin levels directly contributes to mortality. As for angiotensin II, it is known that it promotes cardiac remodeling, and aldosterone may increase myocardial fibrosis - a determinant in the origin of diastolic dysfunction - and cardiac necrosis [10], acting as an endogenous bioactive factor involved in the process of vascular calcification [29]. One group of researchers even found a reduction in mortality in elderly individuals with diastolic dysfunction and CKD with the administration of angiotensin II receptor blockers or the use of angiotensin-converting enzyme inhibitors [30]. The activation of this system conditioned by the loss of ovarian production of estrogens may also be the basis of the pathogenesis of diastolic cardiac dysfunction in postmenopausal women [31]. The presence of moderate CKD culminates in early cardiac fibrosis, moderate diastolic dysfunction, and preserved systolic function [32]. One of the underlying causes of both myocardial stiffness increase and the appearance of fibrotic tissue is the intersticial deposition of collagen in the extracellular matrix. As a matter of fact, patients with abnormal diastolic function have an increased collagen content in myocardial tissue (integrated backscatter) [33]. The relationship between the heart and the kidney is so complex that any alteration in one of them necessarily affects the other in such a way that impaired renal function may more effectively predict left ventricular diastolic dysfunction compared to myocardial ischemia [34]. 

Impact of phospho-calcium (Ca-P) metabolism

The development of diastolic dysfunction in patients with chronic kidney disease may result from disorders of (Ca-P) metabolism, including hypocalcemia, decreased levels of calcitriol, and increased levels of serum phosphorus and parathyroid hormone (PTH). These changes are often associated, especially in the cardiovascular system, implying that disturbances in this metabolism are an implicit factor in the etiopathogenesis of diastolic dysfunction in this group of patients. Hyperphosphatemia is positively correlated with uremia, which is responsible for the progression of vascular calcifications (synthesis of calcium phosphates - hydroxyapatites). Recent studies indicate that diastolic dysfunction negatively affects left ventricular systolic velocity [35,36], which suggests impaired cardiac contractility and a preclinical stage of heart failure development due to reduced mitral annulus systolic velocity. There have even been clinical observations of idiopathic dilated cardiomyopathy caused by hypocalcemia with consequent transient systolic and diastolic left ventricular dysfunction [37]. However, the effect of hypocalcemia on cardiac myocytes is not clearly known, although it is known that the main clinical symptoms of this condition are essentially related to nervous and muscle hyperactivity [38]. According to one study, hypocalcemia is an independent predictive factor for left ventricular diastolic dysfunction in patients with chronic kidney disease [39]. The mechanisms underlying disorders in calcium distribution are not yet well understood due to the complex nature of the physiological processes involved in the regulation of this mineral, especially in patients with CKD. In fact, there is no consensus regarding the efficacy of reducing cardiovascular risk in these patients by using calcium supplementation, and there have even been studies that indicate that the effect of this measure would be practically null [39,40]. According to another study, it could even increase cardiovascular risk [41]. In addition, left ventricular diastolic dysfunction can also be influenced by acid-base balance and administration of calcium (Ca carbonic) preparations, which not only increase serum calcium levels but also promote an increase in pH. 

Vitamin D

Vitamin D has very peculiar biological effects in addition to its contribution to calcium and bone homeostasis. Increasingly, evidence indicates that individuals with 25-hydroxyvitamin D (25(OH)D) deficiency have an increased risk of developing various cardiovascular pathologies such as hypertension, peripheral vascular disease, myocardial infarction, diabetes mellitus, heart failure, and even death [42]. In recent studies, it has been described that the direct effect of vitamin D on cardiomyocytes consists essentially in the acceleration of myocardial relaxation, leading to the assumption that it exerts a determining effect on diastolic function. [43]. High levels of 25(OH)D may also be inversely correlated with the presence of inflammatory markers (C-reactive protein and fibrinogen), being another potential mechanism of its intervention in the reduction of cardiovascular risk. Vitamin D may then have anti-inflammatory and immune-modulating properties, as it exerts an inhibitory effect on renin levels. It was reported in a study that the presence of decreased levels of vitamin D is related to cardiac remodeling phenomena in men of different ethnic groups [44]. It was also shown in another study that vitamin D deficiencies and high CRP values are independently related to the prevalence of diastolic cardiac dysfunction [45]. Other mechanisms associated with the genesis of this pathological condition seem to be associated with cardiac hypertrophy and collagen deposition in myocardial tissue, although there is a study that argues the opposite by proposing that vitamin D leads to increased FGF23 levels and, consequently, to a higher incidence of left ventricular hypertrophy [46]. In a recently published article, it was concluded that vitamin D may act as an important complement to the classic treatment of heart failure since replenishing its values in the event of a deficit may lead to a reduction in aldosterone levels [47]. Subsequent studies are needed to confirm whether there is indeed an association relationship between increased vitamin D levels and decreased cardiovascular risk, namely associated with diastolic dysfunction. 

## Conclusions

Currently, both heart failure with preserved ejection fraction and chronic kidney disease are very prevalent and are closely linked with several other factors, including disturbances in phospho-calcium metabolism and variations in serum vitamin D levels. Although the concept of heart failure began to be explored a few decades ago, further studies are required to better understand the etiopathogenesis of HFpEF, which can be essential to the development of more effective treatments and better outcomes.
